# The dynamic causality between Chinese and ASEAN stock markets

**DOI:** 10.1016/j.heliyon.2023.e22975

**Published:** 2023-12-01

**Authors:** Qingqiao Huang, Mulan Li, Bin Wang

**Affiliations:** a School of Mathematics and Statistics, Guilin University of Technology, Guilin, China; b Guangxi Colleges and Universities Key Laboratory of Applied Statistics, Guilin, China

**Keywords:** COVID-19, Chinese stock market, ASEAN stock markets, Granger causality test, Rolling window

## Abstract

COVID-19 has caused severe shocks to the Chinese and ASEAN stock markets. This paper investigates the relationship between the Chinese and ASEAN stock markets using the bootstrap rolling-window causality test. The results show that there is a bidirectional Granger causality relationship between the Chinese and ASEAN stock markets with time-varying characteristics. Before the COVID-19 outbreak, the interaction between the Chinese and ASEAN stock markets was mainly positive. After the COVID-19 outbreak, during the off-peak period, the interaction between the Chinese and ASEAN stock markets was positive or negative at different periods; during the peak period of the epidemic, the ASEAN stock markets had negative impacts on the Chinese stock market. In addition, the relationship between the Chinese and ASEAN stock markets was enhanced during COVID-19. According to the interaction mechanism, economic and political factors would affect the relationship between the Chinese and ASEAN stock markets, but major events such as COVID-19 have a greater impact. Therefore, macroeconomic policy should play a positive role in the stock market.

## 1 Introduction

In December 2019, the COVID-19 epidemic broke out and spread rapidly around the world, posing a serious threat to human life and health and the economic development of all countries. At the same time, global financial markets had also suffered severe losses, experiencing unprecedented financial turmoil since the 2008 financial crisis [1]. During COVID-19, inter-country risk transmission had accelerated, external shocks have increased the intensity of financial markets in various countries, and the correlation between stock markets had increased [2]. The impact of the epidemic on emerging markets had been great, and the world had paid close attention to it. Compared with emerging markets in Europe, the epidemic had had the greatest impact on emerging markets in Asia [3].

Despite being emerging markets, China and ASEAN are at the forefront of the global response and post-pandemic economic recovery, and China and Vietnam are among the few countries to achieve positive economic growth in 2020 [4]. In view of the remarkable development of China's economy, China's influence in the world is increasing day by day, and the Chinese stock market is also playing an increasingly important role in the stock market in the Asia-Pacific region [5]. On December 31, 2015, ten Southeast Asian countries jointly announced the establishment of the ASEAN Economic Community (AEC), and ASEAN economic integration entered a new stage [6]. Following the rise of China, ASEAN economies are also growing stronger. With the signing of the “Belt and Road” cooperation document and the Regional Comprehensive Economic Partnership Agreement, economic and trade exchanges between China and ASEAN have become increasingly close, and regional economic integration has deepened [7]. In this context, cross-border financial connectivity between China and ASEAN countries continues to develop [8].

Despite the deepening of financial cooperation between China and ASEAN countries, there are significant differences between the financial markets. China's Shanghai Stock Exchange was established in 1990 and has grown to become the third-largest stock exchange in the world by market capitalization [9]. Based on the percentage of stock market capitalization to GDP in 2020, the Singapore stock market ranks first among ASEAN stock markets, followed by Malaysia, Thailand, India, the Philippines, Vietnam, and Indonesia [10]. Over the past two decades, the market capitalization of stock markets in China and Southeast Asia has increased significantly [11]. However, in early 2020, COVID-19 had a huge impact on the Chinese stock market, and China's Shanghai Composite Index fell 13.78 % [12]. ASEAN equity markets have not been spared, with risks in ASEAN financial markets rising sharply at the end of 2019 and intensifying further in 2020. ASEAN stock markets fell sharply, with the Manila Composite Index in the Philippines, the Jakarta Composite Index in Indonesia, and the Straits Index in Singapore down 38 %, 37 %, and 27 % respectively. The combined stock markets of Indonesia, the Philippines, Thailand, and Vietnam lost about a quarter of their value [13].

In recent years, the relationship between China and ASEAN has gradually attracted the world's attention. More and more scholars have studied the connection between the Chinese stock market and the ASEAN stock markets [[14], [15], [16]]. Since the establishment of the China-ASEAN Free Trade Area in 2010, the correlation between China and the five ASEAN countries has been increasing [17]. In addition, the interdependence of the stock market will also increase when major events occur [[18], [19], [20]]. Related studies on the integration of ASEAN stock markets have also gradually increased, and ASEAN stock markets are developing in the direction of more integration [[21], [22], [23], [24], [25]]. However, there is little literature on the relationship between Chinese stock markets and ASEAN stock markets in the context of COVID-19. In addition, almost no scholars use the stock indexes of all ASEAN countries to conduct research, they mostly study the stock markets of ASEAN 5 countries. This paper attempts to fill this gap.

It is of great practical significance to study the interaction between the Chinese and ASEAN stock markets in the context of the COVID-19 pandemic, which can help regulators and investors make better decisions to hedge risks when encountering major events. The purpose of this study is to explore the dynamic interaction between the Chinese stock market and the ASEAN stock markets in the context of COVID-19. Firstly, this paper regards the stock markets of the 10 ASEAN countries as a whole and then adopts the bootstrap rolling-window causality test method to analyze the dynamic causality between the Chinese stock market and the whole stock markets of the 10 ASEAN countries. This paper specifically analyzes whether there is a causal relationship between them, and if so, whether the causal relationship between them will change during COVID-19, whether the causal relationship is unidirectional or bidirectional, positive or negative, etc., and conducts a robustness test for the results.

This paper is structured as follows: Section 2 reviews the relevant literature. Section 3 gives the interaction mechanism between the Chinese stock market and the ASEAN stock markets. Section 4 introduces the methodological theory. Section 5 provides data description and empirical analysis. Section 6 summarizes the full paper.

## 2 Literature review

The relationship between international stock markets has been a hot topic of academic research. Most of them only explored the stock markets of the ASEAN-5 countries (Singapore, Malaysia, Indonesia, Thailand, and the Philippines). Tuan et al. [17] studied cross-country stock market correlation and found that the correlation between China and ASEAN-5 countries was the smallest compared to the U.S. and Japan. However, since the establishment of the China-ASEAN Free Trade Area in 2010, the correlation between China and the ASEAN-5 countries has been increasing. Chien et al. [14] used the recursive cointegration method to explore the convergence of the dynamic process between China and ASEAN stock markets and found that the regional financial integration between China and the ASEAN-5 countries had gradually increased, in addition, there were structural changes in the cointegration relationship between the above six stock markets. Hooy et al. [15] analyzed the degree of stock market response of ASEAN-5 countries to the three major stock market trading reforms in China and obtained that the stock market reforms in China had significant effects on the stock markets in Indonesia, Malaysia, and Singapore. Li and Cai [16] studied the process of stock market integration between China and ASEAN-5 countries and its time-varying characteristics, based on the DCC-GARCH model. The results showed that the dynamic correlation coefficients between China and ASEAN-5 countries’ stock markets increased significantly, except for Thailand, where China had significant time-varying characteristics of financial integration with the other four countries. Kang et al. [26] used multivariate DECO-GARCH models and spillover index to study the dynamic spillover between ASEAN-5 stock markets and world stock markets. They found that there was a positive equi-correlation between ASEAN-5 stock markets and world stock indices, which was more pronounced during the financial crisis, in addition, there was a tendency for the return and volatility spillover to increase during periods of financial crisis. Lee and Jeong [18], Li and Zeng [19], and Caporale et al. [20] studied the impact of the financial crisis on the linkage between the financial markets of the ASEAN-5 countries and China and the United States by GARCH risk decomposition models, time-varying Copula functions and recursive cointegration methods, respectively, they found that the ASEAN-5 countries have become more interconnected with China and the United States. Yiu and Tsang [27] compared the impact of COVID-19, the financial crisis, and the monetary policy tightening scare on the stock markets of ASEAN-5 countries and found that COVID-19 has the most significant impact on ASEAN stock markets. Kamaludin et al. [28] studied the correlation between ASEAN-5 countries and the U.S. Dow Jones index under COVID-19, the stock markets of all ASEAN-5 countries show a strong consistency with the Dow Jones index in the middle of the epidemic development.

Most of the existing studies focus on the stock markets of ASEAN-5 countries, and a few studies discuss the stock markets of ASEAN-6 countries (adding Vietnam). Lean et al. [29] examined the relationship among the stock markets of ASEAN-6 countries and the degree of integration between the stock markets of ASEAN-6 countries and China and found that there is a long-run cointegration relationship between ASEAN-6 countries and China. A shock to one market has a rapid impact on other markets in the short term. Duong and Huynh [30] used non-parametric and parametric (traditional copula and time-varying copula with t-student distribution) methods to study the dependence structure of stock indices in ASEAN countries and found that the Vietnam stock market had the lowest dependence among stock markets of other ASEAN countries and was less affected during the financial crisis. Chen and Wang [12] used the Copula-TV-GARCH-CoVaR model and the MES model to analyze the dependence and risk spillover effects between the stock markets of China and the ASEAN-6 countries. They found that China and Singapore had the highest stock market dependence and Vietnam had the lowest dependence on the Chinese stock market, in addition, there were bidirectional asymmetric risk spillover effects between the stock markets of China and the ASEAN-6 countries. Sadiq et al. [31] studied the impacts of COVID-19 on emerging stock markets in ASEAN-7 countries (adding Myanmar) based on an ST-HAR-type Bayesian posterior model and found that stock markets in Indonesia and Singapore were most severely affected by the epidemic and COVID-19 fear was the ultimate cause of public concern about stock market volatility. In general, with the rapid development of international economic integration, the international financial market has gradually shown a trend of integration, the correlation between global stock markets has been increasing, and major events have further improved the correlation between stock markets.

Many scholars have made important contributions to the field of stock market correlation research, and the research methods they used are mainly cointegration tests, GARCH family models, Copula functions, VAR models, Granger causality tests, etc. In this paper, we use the bootstrap rolling-window causality test to investigate the dynamic causality between the Chinese stock market (CS for brevity) and the ASEAN stock markets (AS for brevity) in the context of COVID-19. Under the framework of time-varying analysis, this research method not only captures more structural change information but also provides a more comprehensive picture of the dynamic impact between CS and AS in different time intervals.

## 3 The interaction mechanism between CS and AS

### 3.1 The influence mechanism of CS on AS

The economic base hypothesis suggests that changes in national macroeconomics can cause stock market fluctuations and that linkages in the economies of countries with the same macroeconomic variables will lead to linkage effects in the stock markets of these countries as well [32]. The macroeconomic variables include trade, foreign direct investment, and currency, etc.

China's macroeconomy has a significant impact on the financial integration between China and ASEAN, where trade is an important determinant [33]. 2022 is the opening year of the comprehensive strategic partnership between China and ASEAN, with closer economic and trade ties and ASEAN continuing to maintain its position as China's top trading partner. In this year, the total value of trade between China and ASEAN reached $975.34 billion, an increase of 11.2 %, of which, China's exports to ASEAN were $567.29 billion, an increase of 17.7 %, and China's imports to ASEAN were $408.05 billion, an increase of 3.3 % [34]. China's trade policy is a factor in the ASEAN market that has a significant impact on regional systemic risk [35].

The establishment of the China-ASEAN Free Trade Area has had a significant positive impact on foreign direct investment (FDI) in member countries [36]. FDI helps host countries to accumulate capital and promote the development of financial markets [37]. Despite the ongoing impact of the pandemic, FDI inflows to ASEAN increased by 42 % to $174 billion in 2021, returning to pre-pandemic levels, with Chinese investment in ASEAN increasing by 96 % to nearly $14 billion. ASEAN remains the largest FDI destination in the developing region after China [38].

The monetary policy of CNY affects the correlation between China and ASEAN stocks. At the beginning of the internationalization of the CNY, the dynamic convergence of the stock markets of China and ASEAN countries showed a trend of strengthening [39]. The increase in the internationalization of CNY has a significant positive impact on the integration of China-ASEAN stock markets [40].

Many econometric models are multiplier models [41,42], and this paper draws on [43] to hypothesize the following mechanism for the impact of CS on AS industry.

AS=T*F*MC

where AS represents the ASEAN stock markets, T denotes the trade between China and ASEAN, F denotes FDI between China and ASEAN, and MC denotes the monetary policy of CNY.

### 3.2 The influence mechanism of AS on CS

ASEAN is gaining prominence on the world stage, with its huge market potential and geographical proximity to China, making it a vital partner for China. On November 22, 2021, at the 30th-anniversary summit of China-ASEAN dialogue relations, President Xi Jinping officially announced the upgrading of China-ASEAN relations to a comprehensive strategic partnership [44]. The increasingly close relationship between China and ASEAN will also generate some correlation between stock markets. AS can influence CS through factors such as geopolitics and industrial transfer.

After long-term international exchanges, China and the United States have formed close ties with ASEAN in terms of politics, economy, culture, and other aspects, and have substantial geopolitical interests in ASEAN. Moreover, the geopolitical influence of the two countries in ASEAN is constantly increasing [45]. In recent years, under the influence of anti-globalization and COVID-19, China has proposed to build a new development pattern in which domestic and international dual circulation promote each other. The financial cooperation between China and ASEAN has been continuously deepening. However, the implementation of the “Asia-Pacific rebalancing” strategy by the United States will have an impact on the cooperative relationship between China and ASEAN [46]. Geopolitical risk has a significant nonlinear Granger causality relationship with China's stock market [47].

In 2015, the Chinese government put forward the strategic task of transformation, upgrading, and innovative development of China's manufacturing industry in the “13th Five-Year Plan” [48]. And in recent years with the appreciation of CNY and the increase of labor costs in China in recent years, the labor costs in ASEAN countries still remain low. So ASEAN is relatively more competitive in attracting foreign investment in manufacturing, thereby prompting enterprises from various countries to transfer labor-intensive industries in China to ASEAN countries. In addition, other factors such as resource endowment, market size, and geographical location are also motivating factors for Chinese investment in ASEAN [49]. The overseas development of China's “going out” enterprises cannot be separated from the financial support of the host country, and the financial development of the host country plays a role in promoting industrial transfer [50].

**Fig. 1:**
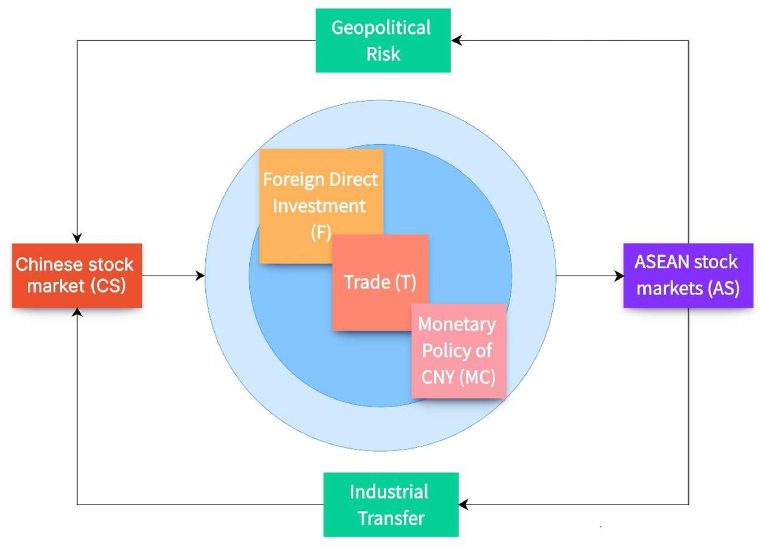
Interaction mechanism between CS and AS.

In summary, the interaction mechanism between CS and AS can be obtained (Fig. 1).

## 4 Methodology

### 4.1 Bootstrap full-sample causality test

The Bootstrap rolling-window causality test method is based on the traditional Granger causality test and uses the rolling-window to test the causality of the whole sample data and the sub-sample data respectively. The traditional Granger causality test determines the causality of the object of study by calculating *Wald*, likelihood ratio (*LR*), and Lagrange multiplier (*LM*) statistics. However, the reliability of traditional methods may be reduced in the case of small samples [51]. Shukur and Mantalos [52,53] found that the residual-based bootstrap technique (*RB*) and the modified *LR* statistic based on the bootstrap technique had the best test strength and adaptability. They developed the critical value of the *RB* method to avoid result bias and improve the accuracy of the Granger causality test. In addition, they argue that this method is suitable for testing standard asymptotic distributions, even for small samples, and that the *LR* test can be modified by the characteristics of power and size.

The bivariate VAR (*p*) model is constructed as follows:

(1) $$y_{t}=\varphi_{0}+\varphi_{1}y_{t-1}+\cdots +\varphi_{p}y_{t-p}+\varepsilon_{t},t=1,2,3,\ldots \text{,}T$$

where $y_{t}$ is the k dimensional endogenous variable, T refers to the number of samples, $\varepsilon_{t}$ is a white noise process with zero mean, and p is the optimal lag order determined by the Akaike information criterion (AIC) or the Schwartz information criterion (SIC). Variable $y_{t}$ is divided into $y_{t}={\left(y_{1t},y_{2t}\right)}^{\prime }$, Eq. (1) can be rewritten as:

(2) $$\left[\begin{array}{c} y_{1t}\\ y_{2t} \end{array}\right]=\left[\begin{array}{c} \varphi_{10}\\ \varphi_{20} \end{array}\right]+\left[\begin{array}{cc} \varphi_{11}\left(L\right) & \varphi_{12}\left(L\right)\\ \varphi_{21}\left(L\right) & \varphi_{22}\left(L\right) \end{array}\right]\left[\begin{array}{c} y_{1t}\\ y_{2t} \end{array}\right]+\left[\begin{array}{c} \varepsilon_{1t}\\ \varepsilon_{2t} \end{array}\right]\text{.}$$

here $y_{1t}$ and $y_{2t}$ represent the variables of CS and AS respectively. $\varphi_{ij}\left(L\right)=\sum_{k=1}^{p}\varphi_{ij,k}L^{k},i,j=1,2$, L is the lag operator and $L^{k}y_{t}=y_{t-k}$.

Specifically, assume the null hypothesis that CS is not the Granger cause of the change in AS as $\varphi_{21,k}=0\;\left(k=1,2,\cdots ,p\right)$, CS had significant effects on AS when the null hypothesis was rejected; the null hypothesis that AS is not the Granger cause of the change in CS as $\varphi_{12,k}=0\;\left(k=1,2,\cdots ,p\right)$. AS had significant effects on CS when the null hypothesis was rejected.

### 4.2 Parameter stability test

There is a prerequisite for the full-sample Granger causality test, that is, it is assumed that the parameters of the VAR model are stable and there is no structural change, but in reality, the time series is likely to have structural mutations during the sample period [54]. Structural mutations will lead to unstable or inaccurate causal relationships [55]. Andrews [56] and Andrews and Ploberger [57] proposed three statistics, *Sup-F*, *Ave-F*, and *Exp-F* to test the stability of model parameters in the short run. *Sup-F* statistic is used to test for structural changes in the parameters, and *Ave-F* and *Exp-F* statistics are used to test for changes in the parameters over time. In addition, the three statistics *Sup-F*, *Ave-F*, and *Exp-F* require a bilateral 15 % correction to the test sample. Therefore, the actual sample interval for the parameter stability test is (0.15, 0.85). In addition, this paper uses the $L_{c}$ statistics proposed by Nyblom [58] and Hansen [59] to test whether the parameters follow the random walk process to test the long-term stability of the parameters. If the model parameters are unstable, further tests are needed.

### 4.3 Bootstrap sub-sample rolling-window causality test

In order to overcome the problem of parameter instability, Bilcilar et al. [60] proposed the bootstrap sub-sample rolling-window causality test. This method divides the whole sample into many subsamples according to the fixed window width and then lets the subsamples roll from the first end to the end of the whole sample sequence. Let the length of the time series be T, and the length of the sub-sample be l, and the sample period of any sub-sample be $\tau -l+1\text{,}\tau -l,\cdots \text{,}T\begin{array}{cc} , & \left(\tau =l,l+1,\cdots ,T\right) \end{array}$, then the full sample becomes a sequence of T−l sub-samples. This can reflect the time-varying characteristics of the causal relationship between CS and AS. In addition, the method can quantify the degree of interaction between CS and AS. Let $N_{b}$ denote the number of bootstrap repetitions, ${\hat{\varphi }}_{12,k}^{*}$ and ${\hat{\varphi }}_{21,k}^{*}$ be the estimates of the VAR model in Equation (2). Then $N_{b}^{-1}\sum_{k=1}^{p}{\hat{\varphi }}_{21,k}^{*}$ is the degree of influence of CS on AS, and $N_{b}^{-1}\sum_{k=1}^{p}{\hat{\varphi }}_{12,k}^{*}$ is the degree of influence of AS on CS. This paper uses the 90 % confidence interval, and the upper and lower bounds of the impact coefficients are the 95th and 5th quantiles in ${\hat{\varphi }}_{12,k}^{*}$ and ${\hat{\varphi }}_{21,k}^{*}$, respectively.

The selection of scroll window width should not only consider model accuracy but also consider the representativeness of test statistics. Bilcilar et al. pointed out that the accuracy of the rolling-window test mainly depends on the window width. Although a larger window width can improve the model accuracy, heterogeneity may affect the accuracy of the results, thus reducing the representativeness of the test statistics. Although a smaller window width can improve the representativeness of test statistics, it also reduces the test accuracy. Bootstrap technology can be used to solve this problem to obtain better accuracy. Pesaran and Timmerman [61] found that in the case of structural mutation, the selection of the optimal window size depends on the durability and size of the fracture, and when there are multiple structural change points, the minimum window size is limited to 20. In this paper, the window width is set to 20–50 for many simulations, and the more appropriate window width is finally selected as 20.

## 5 Empirical data and analysis

This paper selects China-related stock indice and ASEAN-9 countries-related stock indices of Malaysia, Indonesia, Thailand, the Philippines, Singapore, Vietnam, Laos, Myanmar and Cambodia for empirical analysis, excluding Brunei because there is no stock exchange in Brunei at present. The CSI 300 index is used to represent CS. It consists of the 300 most representative securities of large size and liquidity in the Shanghai and Shenzhen markets, which can reflect the overall performance of listed stock prices in the Chinese A-share market in a comprehensive manner. GDP is a good indicator that can measure investment and stock markets [62,63] and is a good measure of ASEAN's leading indicator of stock market movements [64]. Considering the integrity of the ASEAN stock markets, the daily returns of representative stocks of each country are weighted by the share of GDP of each country in each year to construct the total ASEAN stock returns. Relevant stock indexes of ASEAN countries are as follows: Malaysia's KLCI Index, Indonesia's JKSE Index, Thailand's SET Index, the Philippines' PSEi Index, Singapore's STI Index, Vietnam's VNINDEX Index, Laos' LSX Index and Myanmar's MYANPIX Index and Cambodia's CSX Index. The formula for calculating the return rate of the stock index is: $R_{it}=\ln\;P_{it}-\ln\;P_{it-1}$, where $P_{it}$ is the closing prices of the economy i on t day. Myanmar officially started trading on March 25, 2016, making it the latest of the nine ASEAN countries to trade. In addition, taking the two important periods around the COVID-19 outbreak into account, we take daily stock data from March 28, 2016, to December 27, 2022, excluding non-common trading day data, and obtain 1145 sets of sample data. The data for Laos stocks are from the official website of the Lao Stock Exchange (http://www.lsx.com.la/), and for Myanmar stocks from the official website of the Yangon Stock Exchange (https://ysx-mm.com/). Cambodia stock data from the Cambodia stock exchange's website (http://csx.com.kh/main.do), and the rest of the stock data are derived from the Wind database, the GDP figures come from the International Monetary Fund (IMF) database.

### 5.1 Descriptive statistical analysis

**Fig. 2:**
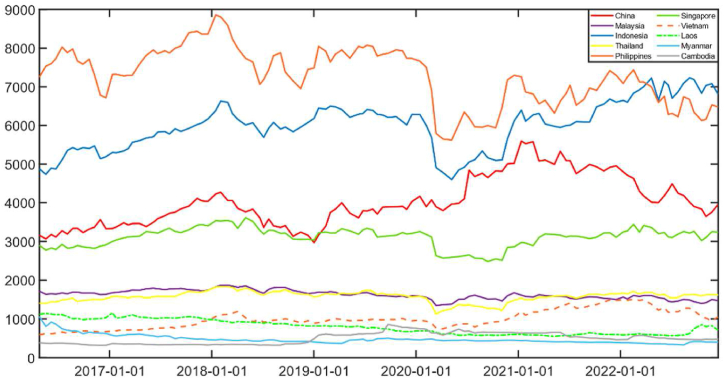
China and ASEAN countries stock index.

Fig. 2 shows that after the outbreak of COVID-19, the stock markets of China and ASEAN countries have been affected to varying degrees. Relatively speaking, stock markets in China, Indonesia, the Philippines, and Singapore were more volatile, with stock prices falling severely. It was not until early 2021 that these stock markets returned to pre-pandemic levels, indicating that the impact of the epidemic on the stock markets of these countries is large and persistent. The stock market volatility in Malaysia, Laos and Myanmar are relatively flat, indicating that the epidemic has affected stock markets in these countries to a lesser extent. Overall, Indonesia and the Philippines has the most similar stock market movements, while Malaysia, Thailand and Singapore have the most similar stock market fluctuations.

Descriptive analysis is shown in Table 1. According to the results of the ADF test, both CS and AS reject the null hypothesis of the existence of unit roots at the significance level of 1 %, indicating that both time series are stable. The skewness of CS and AS is negative, and the kurtosis is greater than 3, showing the typical characteristics of the “sharp peak and thick tail”. Jarque-Bera statistics show that CS and AS both reject the null hypothesis of Gaussian distribution at the significance level of 1 %, indicating that the stock index return series of China and the stock index return series of ASEAN are subject to non-normal distribution. In addition, the standard deviation of CS is greater than that of AS, indicating that the volatility of CS is greater than that of the whole AS.

**Table 1:** Descriptive statistics.

	CS	AS
Mean	−0.00001	0.00018
Median	0.00035	0.00043
Maximum	0.04226	0.04448
Minimum	−0.08209	−0.06090
Standard Deviation	0.01155	0.00682
Skewness	−0.59917	−1.66066
Kurtosis	6.92716	19.51178
Jarque-Bera	803.5931***	13521.6***
ADF	−35.52313***	−16.24637***

Notes: *** indicates significance at the 1 % levels.

### 5.2 Bootstrap full-sample causality test

The prerequisite of bootstrap rolling-window causality test is stable. The above results show that a model can be established. The results of AIC test and SIC test both show that the optimal lag order is 4. Therefore, this paper establishes the bivariate VAR (4) model of CS and AS, and uses *RB*-modified *LR* statistics for testing. The *LR* statistics and bootstrap-*p* values after testing are shown in Table 2.

**Table 2:** Full-sample test result.

Tests	H_0_：CS is not the Granger reason for AS		H_0_：AS is not the Granger reason for CS	
	Statistics	*p*-values	Statistics	*p*-values
Bootstrap LR test	4.6407	0.3983	11.8694	0.0339

Notes: To calculate *p*-values using 10,000 bootstrap repetitions. ** indicates significance at the 5 % levels.

The results of the full-sample causality test show that there is no bidirectional causality between CS and AS. The null hypothesis that AS is not a Granger cause of CS is rejected, indicating that AS is a Granger cause of CS. With the development of global financial integration and the increasingly close cooperation between China and ASEAN, AS will certainly have some influence on CS. But the results show that CS is not a Granger cause of AS. However, the fact that China has a more significant impact on the ASEAN financial sector due to the rapid expansion of Chinese companies and financial investments around the world, and its strengthened monetary and exchange rate policies [15]. This is most likely due to the structural changes in the parameters of the VAR model, which may affect the accuracy of the full-sample Granger causality test. Therefore, the stability of the model parameters needs to be tested.

### 5.3 Parameter stability test

Table 3 shows the parameter test results of CS, AS and the VAR system. The results of the *Sup-F* test show that the parameters of CS reject the null hypothesis at the significance level of 5 %, and the parameters of AS and the VAR system reject the null hypothesis at the significance level of 1 %, which means that the parameters are considered to have structural changes. *Ave-F* test results show that the parameters of AS and the VAR system are significant at the significance level of 1 %, which means that the parameters have significantly shifted over time, but the parameters of CS did not pass the significance test. Meanwhile, according to the *Exp-F* test results, the parameters of CS, AS, and the VAR system are significant. That is, it is believed that parameters change with time. *Sup-F*, *Ave-F*, and *Exp-F* test results show that the parameters of CS, AS and the VAR system are not stable in the short term. In addition, the *L*_*c*_ test results show that the parameters of the VAR system reject the null hypothesis at the significance level of 1 %, indicating that the parameters do not have long-term stability. In a word, the parameters fail the significance test and are unstable.

**Table 3:** Parameter stability test result.

Tests	CS		AS		VAR system	
	Statistics	*p*-values	Statistics	*p*-values	Statistics	*p*-values
Sup-F	28.8229**	0.0157	33.3014***	0.0032	110.2187***	0.0000
Ave-F	11.9191	0.1441	19.5106***	0.0027	37.9032***	0.0001
Exp-F	8.6496*	0.0818	11.5107**	0.0101	48.8235***	0.0000
L_c_					6.2036***	0.0050

Notes: To calculate *p*-values using 10,000 bootstrap repetitions.

***, ** and * indicate significance at the 1 %, 5 % and 10 % levels, respectively.

### 5.4 Sub-sample rolling-window causality test

According to the test results of parameter stability, Granger causality between CS and AS is not constant, and structural changes exist in the VAR model. Therefore, the bootstrap sub-sample rolling-window causality test are used to study the time-varying Granger causality between CS and AS.

**Fig. 3:**
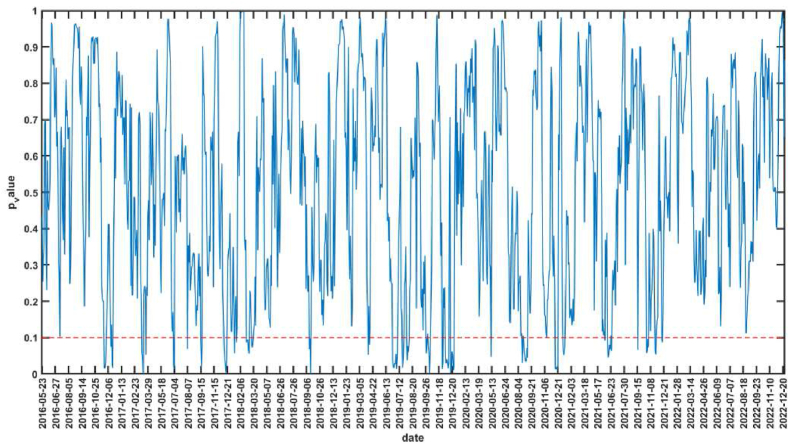
The rolling-window test *p*-value of CS is not Granger cause for AS.

**Fig. 4:**
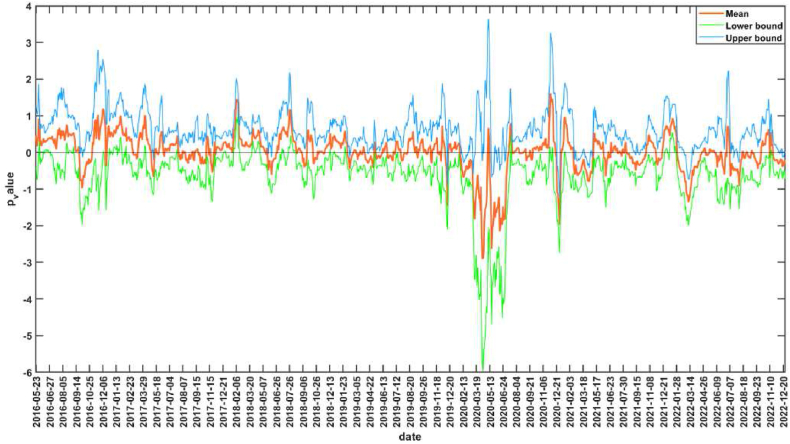
The rolling-window influence coefficient of CS on AS.

The null hypothesis corresponding to the bootstrap-*p* value in Fig. 3 is that CS is not the Granger reason for the AS. The interval of *p* value less than 0.1 indicates that the null hypothesis is rejected at the significance level of 10 %, that is, CS is the Granger reason of AS. The sub-sample intervals that are significant at 10 % level of significance are 2016.11.18–2016.11.29, 2016.12.8–2016.12.16, 2017.12.12–2017.12.21, 2018.9.6–2018.9.18, 2019.6.25–2019.7.5, 2019.7.18–2019.8.14, 2019.9.26–2019.10.30, 2019.11.29–2019.12.11, 2020.8.13–2020.9.15, 2021.6.8–2021.6.24, 2021.10.27–2021.11.25, etc. Fig. 4 shows the mean and upper and lower bounds of the bootstrap rolling-window influence coefficient of CS on AS. If the mean value of the influence coefficient is greater than 0, it indicates a positive impact of CS on AS, and if the mean value is less than 0, it indicates a negative impact of CS on AS. 2016.11.18–2016.11.29, 2016.12.8–2016.12.16 and 2017.12.12–2017.12.21 periods, there is a positive impact of CS on AS. In July 2016, the RCEP between China and ASEAN came into effect [65], and the trade volume between China and ASEAN countries reached a record high in 2017 [66], and the relationship between the two sides continues to develop deeply. At the same time, ASEAN countries had gained wider market space for labor-intensive industries due to China's industrial upgrading, and ASEAN countries were in a key position in the “Belt and Road” construction proposed by China, and cooperation between China and ASEAN in economic, trade, and financial fields had been comprehensively promoted, with China's development driving ASEAN's development [67]. The CS in general showed an upward trend during these periods, and AS in general also showed an upward trend. There was a negative impact of CS on AS during 2018.9.6–2018.9.18 and 2019.6.25–2019.7.5. In June 2018, the Trump administration imposed a 25 % tariff on $34 billion worth of Chinese-made products, triggering the Sino-U.S. trade war. In the early stages of the Sino-U.S. trade war, CS were volatile, while AS was more stable in terms of volatility. However, in mid-to-late 2019, the impact of the Sino-U.S. trade war on ASEAN became increasingly evident, with ASEAN countries overall experiencing a decline in their economic growth ratios as the Sino-U.S. trade war disrupted ASEAN's integral global supply chains. The decline in CS and the overall decline in AS validates the positive impact of CS on AS during 2019.7.18–2019.8.14、2019.9.26–2019.10.30 and 2019.11.29–2019.12.11. After the COVID-19 outbreak, the Chinese government immediately took a series of strong measures to effectively contain the outbreak, and China's accommodative monetary policy effectively maintained financial stability and successfully contributed to the rapid post-crisis economic recovery, enhancing international investors' confidence in China's financial markets [68]. The CS showed a rebound, but ASEAN was still affected by the epidemic and the stock market was sluggish. Therefore, during the periods of 2020.8.13–2020.9.15 and 2021.6.8–2021.6.24, which belong to the off-peak period of the epidemic, there is a negative impact of CS on AS. During the period 2021.10.27–2021.11.25, there is a positive impact of CS on AS. This period is during the off-peak period of the epidemic, when the epidemic is more stable and China is the first major economy in the world to achieve positive growth, many countries, especially Asian and ASEAN countries, will benefit from the strong recovery of the Chinese economy [69], while the loose monetary policy in ASEAN countries helps to mitigate the stock market volatility during the epidemic [27]. In addition, the volatility of the influence coefficient increases significantly after the outbreak with the largest absolute value, indicating that the impact of CS on AS increases significantly during COVID-19, and also has time-varying characteristics.

**Fig. 5:**
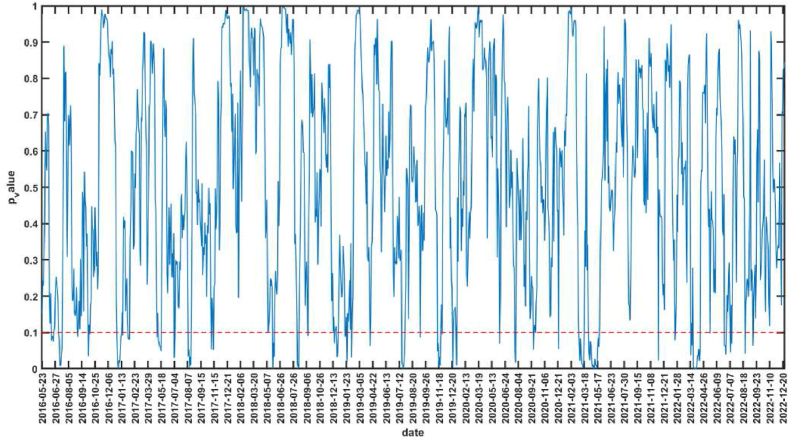
The rolling-window test *p*-value of AS is not Granger cause for CS.

**Fig. 6:**
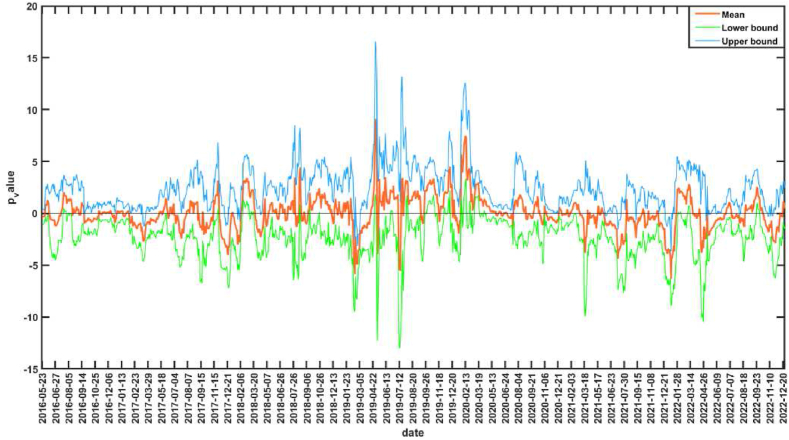
The rolling-window influence coefficient of AS on CS.

The null hypothesis corresponding to the bootstrap-*p* value in Fig. 5 is that AS is not the Granger reason for the CS. The interval of *p* value less than 0.1 indicates that the null hypothesis is rejected at the significance level of 10 %. Fig. 6 shows the mean and upper and lower bounds of the bootstrap rolling-window influence coefficient of AS on CS. 2016.7.15–2016.7.29, 2016.12.27–2017.1.13, 2017.8.7–2017.8.16, 2020.1.8–2020.1.22, 2021.3.9–2021.3.18, 2021.3.25–2021.5.17, 2022.3.23–2022.4.26, these sub-sample intervals are significant at 10 % level of significance. 2016 is the 25th anniversary of China-ASEAN dialogue relations and 2017 is the 50th anniversary of ASEAN and the year of China-ASEAN tourism cooperation [70], the relationship between the two sides continues to develop deeply, AS and CS are both on an upward trend. 2020.1.8–2020.1.22 is in the early stage of the outbreak, CS and AS are turbulent, and AS has a positive impact on CS. During 2021.3.9–2021.3.18 and 2021.3.25–2021.5.17, AS has a negative impact on CS. After the outbreak, China adhered to the “dynamic zero” policy and implemented strict measures to prevent and control the epidemic, and CS is less affected by the epidemic and relatively stable. However, during this period, AS was affected by the epidemic and the stock market fell. 2022.3.23–2022.4.26 was the peak period of both the Chinese and ASEAN epidemic outbreaks, but compared to most of the ASEAN countries, China's epidemic prevention and control measures were stricter, and investors considering CS and AS would prefer the more mature CS, resulting in a negative impact of AS versus CS during this period.

### 5.5 Robustness test

VAR model and its extension form pay more attention to the selection of variables. In order to ensure the reliability of the empirical results, the method [71,72] is used for reference in this paper, and the robustness test is conducted by adding control variables to the model. Twitter's happiness sentiment can be viewed as investor sentiment. Due to the widespread use of the Internet, Twitter's happiness sentiment has been shown to have a significant influence on stock price fluctuations [73]. There are international investors in CS and AS, and investor sentiment will have an impact on CS and AS. Therefore, we chose Twitter's happiness sentiment index as the control variable.

After adding control variables to the model, the results of the Granger causality test for the whole sample, parameter stability test, and bootstrap sub-sample rolling-window causality test are still basically consistent with the original results. We came to the same conclusion as before. Before the outbreak of COVID-19, the interaction between CS and AS was mainly positive. After the COVID-19 outbreak, during the off-peak period, the interaction between CS and AS was positive or negative at different periods; during the peak period of the epidemic, AS had negative impacts on CS. In addition, the relationship between CS and AS is enhanced during COVID-19. Therefore, the empirical results of this paper are robust, and the causal relationship between CS and AS is real and reliable.

## 6 Conclusion

The purpose of this paper is to investigate the dynamic causal relationship between CS and AS in the context of COVID-19. First, the bootstrap full-sample Granger causality test is used for the analysis, and the results show that AS is the Granger cause of CS and CS is not the Granger cause of AS. However, the results of the parameter stability test reveal that there are structural changes in the parameters of CS, AS, and the VAR system, which can affect the accuracy of the test. Therefore, the time-varying correlation between CS and AS is further analyzed by using the bootstrap sub-sample rolling-window causality test, and the following conclusions are finally obtained: First, there is a bidirectional Granger causality between CS and AS, and it has time-varying characteristics. Before the outbreak of COVID-19, China and ASEAN had close cooperation and frequent economic and trade exchanges. The interaction between CS and AS was mainly positive, and their stock markets ran relatively stable. However, there were periods when the CS had negative impacts on the AS due to the Sino-U.S. trade war. After the outbreak of COVID-19, during the off-peak period of the epidemic, CS had positive or negative impacts on AS at different periods. Similarly, AS had positive or negative impacts on CS at different periods, indicating that CS cannot always be regarded as the safe haven of AS, and vice versa. At the peak of the epidemic, AS had a negative impact on CS, indicating that AS was more affected by the epidemic than CS. Second, during COVID-19, the interaction between CS and AS had significantly increased, indicating that the epidemic has had a great impact on their relationship. According to the interaction mechanism, economic and political factors would affect the relationship between CS and AS, but major events such as COVID-19 have a greater impact.

According to the research results, the following enlightenment can be obtained. First, there is a bidirectional Granger causality between CS and AS. Therefore, we can predict the volatility of AS through the changes in CS, and in turn, we can also predict the risks of CS through the risks of AS. For example, regulators and investors in CS can pay attention to changes in the financial policies of ASEAN countries and take corresponding measures promptly to guard against imported risks from other countries. When the stock market is relatively stable, investors should invest rationally, avoid the influence of the “herd effect”, and prevent the linkage effect of bubble risk. Relevant departments should give full play to the positive role of macroeconomic policies, further deepen China-ASEAN regional financial cooperation, and promote China-ASEAN cross-border financial innovation. Second, under the influence of major events such as COVID-19, the correlation between CS and AS has increased and is time-varying. This means that financial risk is becoming more contagious. Regulators should adjust financial policies promptly according to the impact of major events on the stock market and maintain the stable operation of the financial market. Investors should build a reasonable portfolio to diversify their investment risks and not put all their eggs in the same basket. In addition, China and ASEAN should accelerate the formation of a regional multilateral financial regulatory cooperation mechanism, take appropriate macro-prudential measures, and guard against spillover effects of external risks.

Compared with previous studies, this paper studies the dynamic interaction between CS and AS under the background of COVID-19 and extends to the stock markets of ten ASEAN countries to portray AS more comprehensively. However, there are still some shortcomings in this study. First, this study only takes COVID-19 as the background and does not conduct quantitative research on COVID-19. Second, empirical results show that there is a bidirectional dynamic causal relationship between CS and AS, and the causal relationship is significantly enhanced during COVID-19. However, we cannot exclude the possibility that the results of the study may have been influenced by other political, economic, and financial factors. Third, this paper studies the stock markets of all ASEAN countries as a whole and fails to understand in detail the impact on the stock markets of each ASEAN country. As a result, future studies are likely to quantify major events, more accurately portray the impact of major events on the stock market, and conduct a more detailed study of the stock markets of each ASEAN country.

## Funding

This article was supported partially by the 10.13039/501100001809National Natural Science Foundation of China (Nos. 11961015,71963008). The views expressed in this article are those of the authors and do not necessarily represent the views of their affiliated institutions, NFSC, or China's government.

## Data availability statement

Data will be made available on request.

## Additional information

No additional information is available for this paper.

## CRediT authorship contribution statement

**Qingqiao Huang:** Writing – review & editing, Writing – original draft, Validation, Investigation, Formal analysis, Data curation. **Mulan Li:** Writing – review & editing, Software, Resources. **Bin Wang:** Writing – review & editing, Supervision, Project administration, Methodology, Funding acquisition, Conceptualization.

## Declaration of competing interest

The authors declare the following financial interests/personal relationships which may be considered as potential competing interests:Bin Wang reports article publishing charges was provided by National Natural Science Foundation of China. If there are other authors, they declare that they have no known competing financial interests or personal relationships that could have appeared to influence the work reported in this paper.
